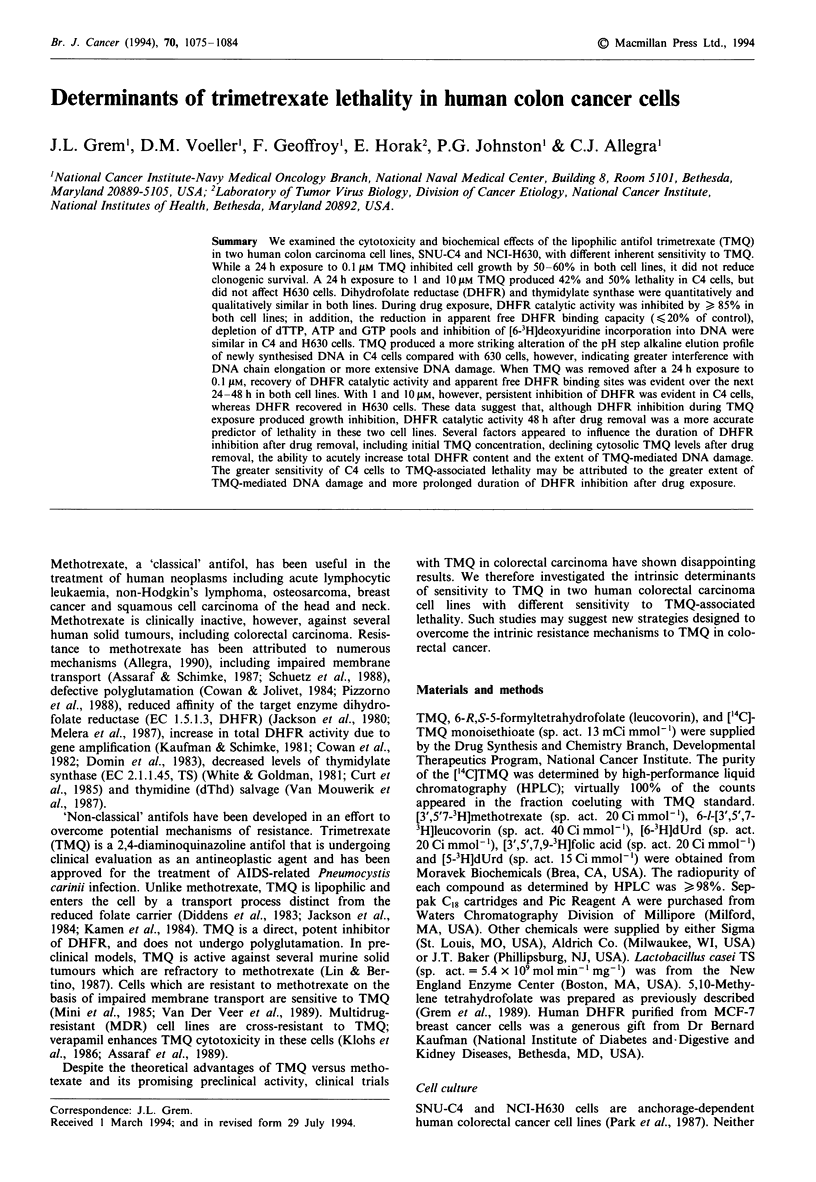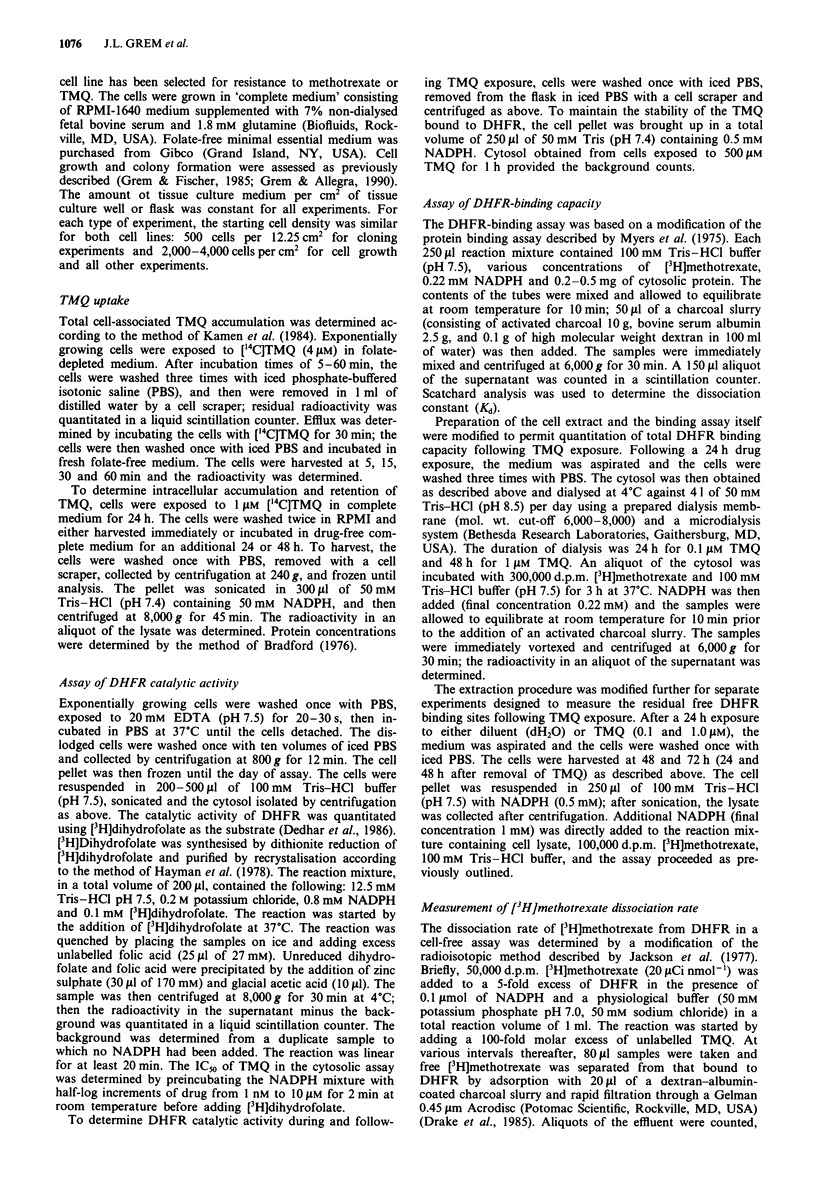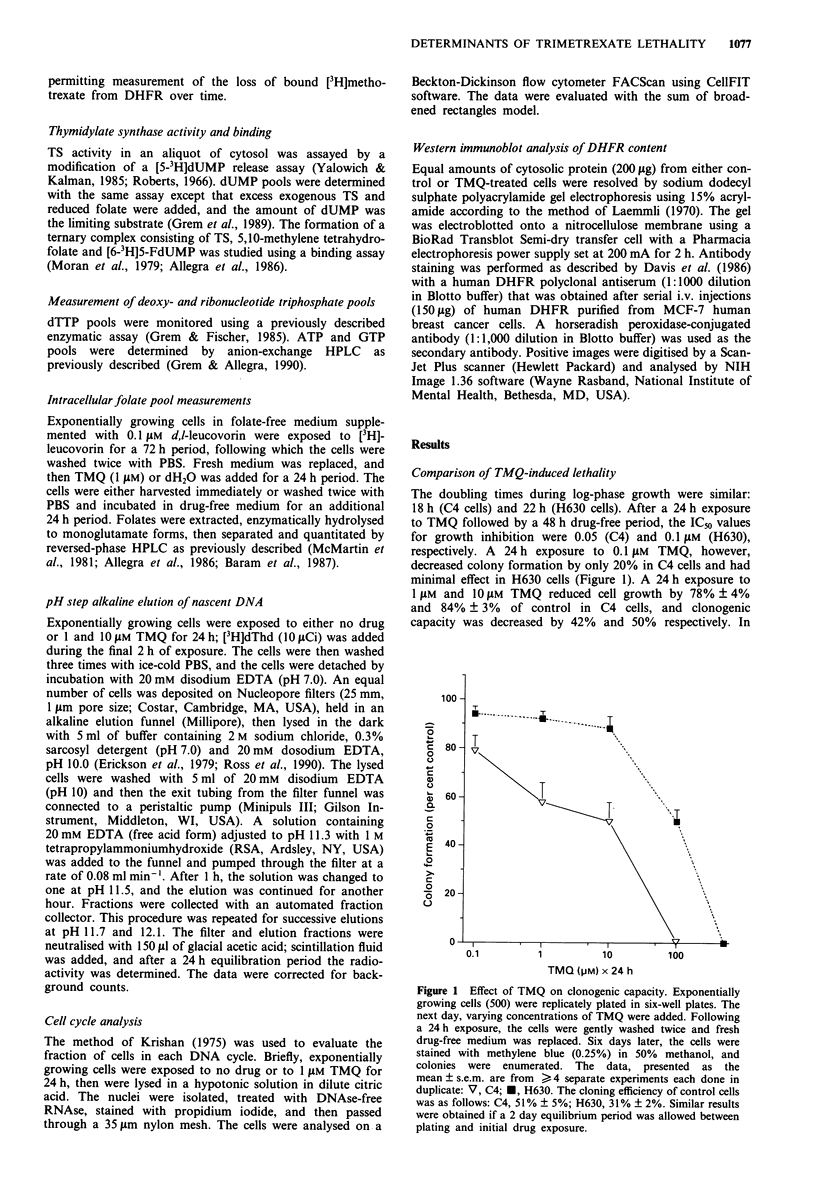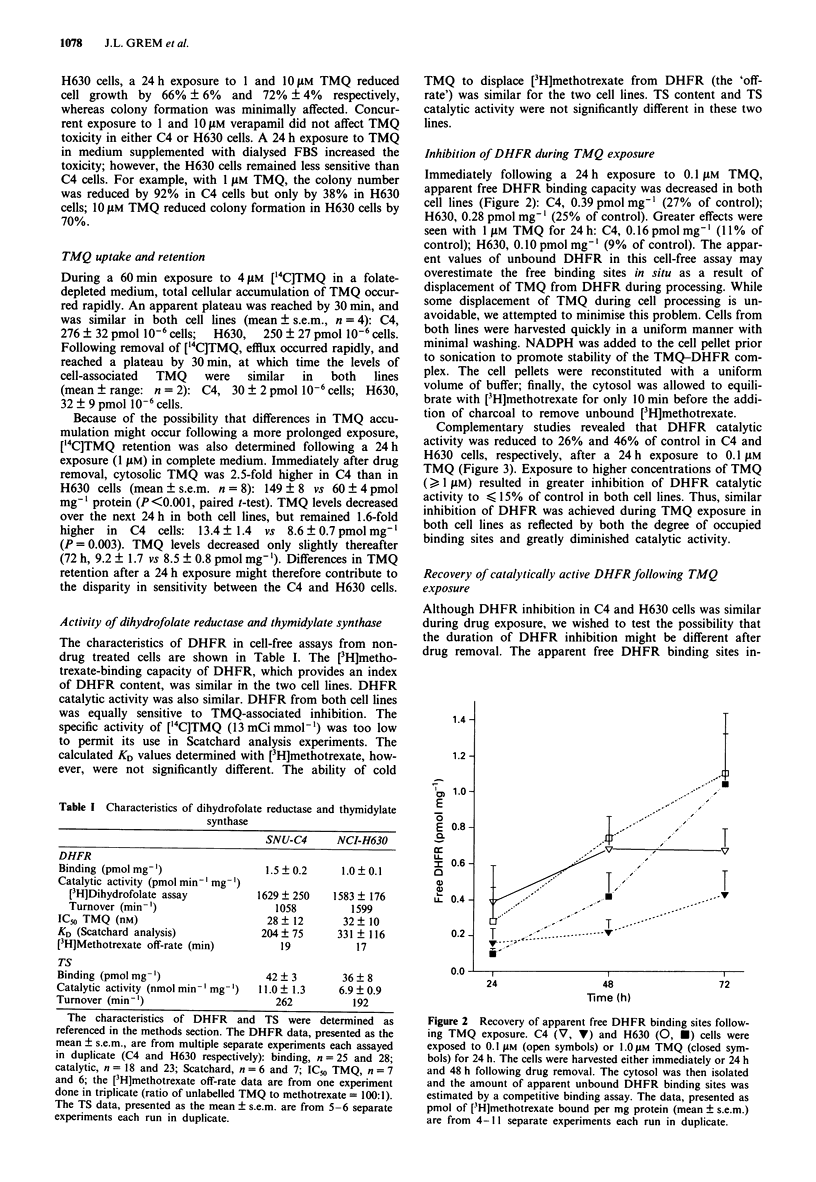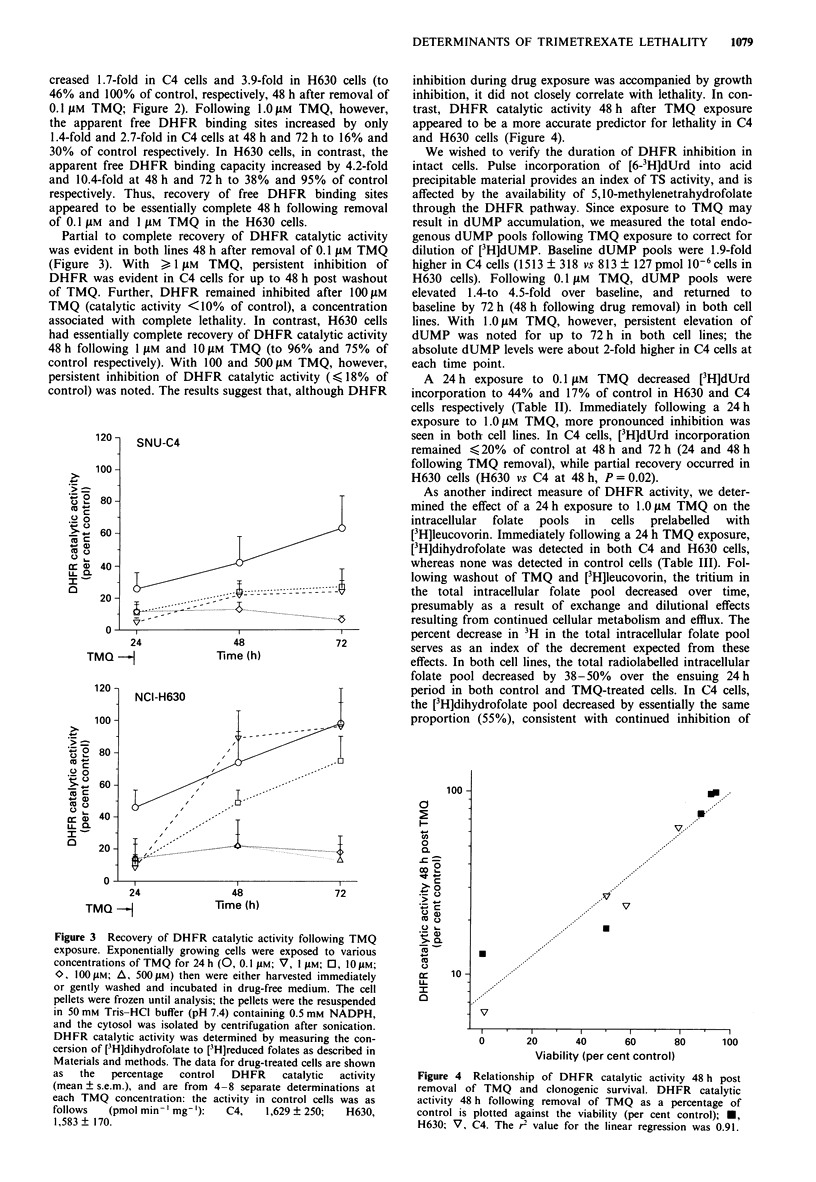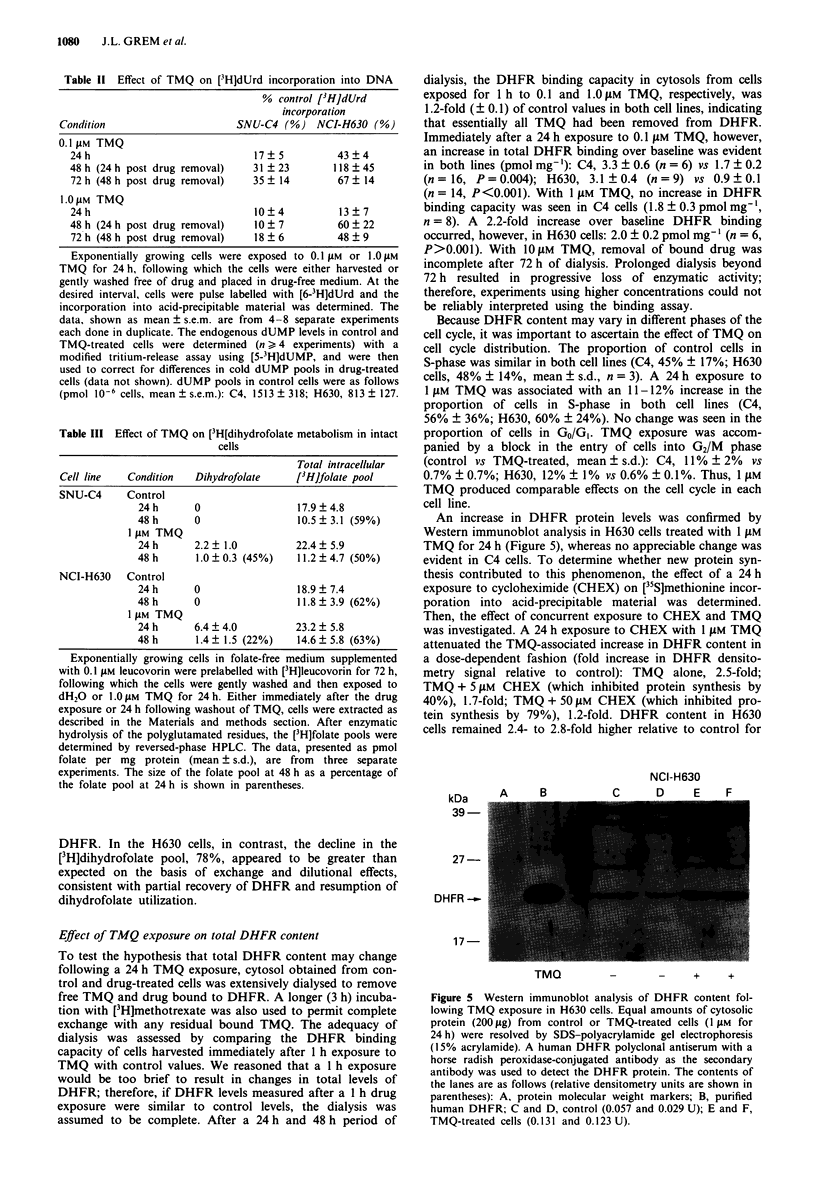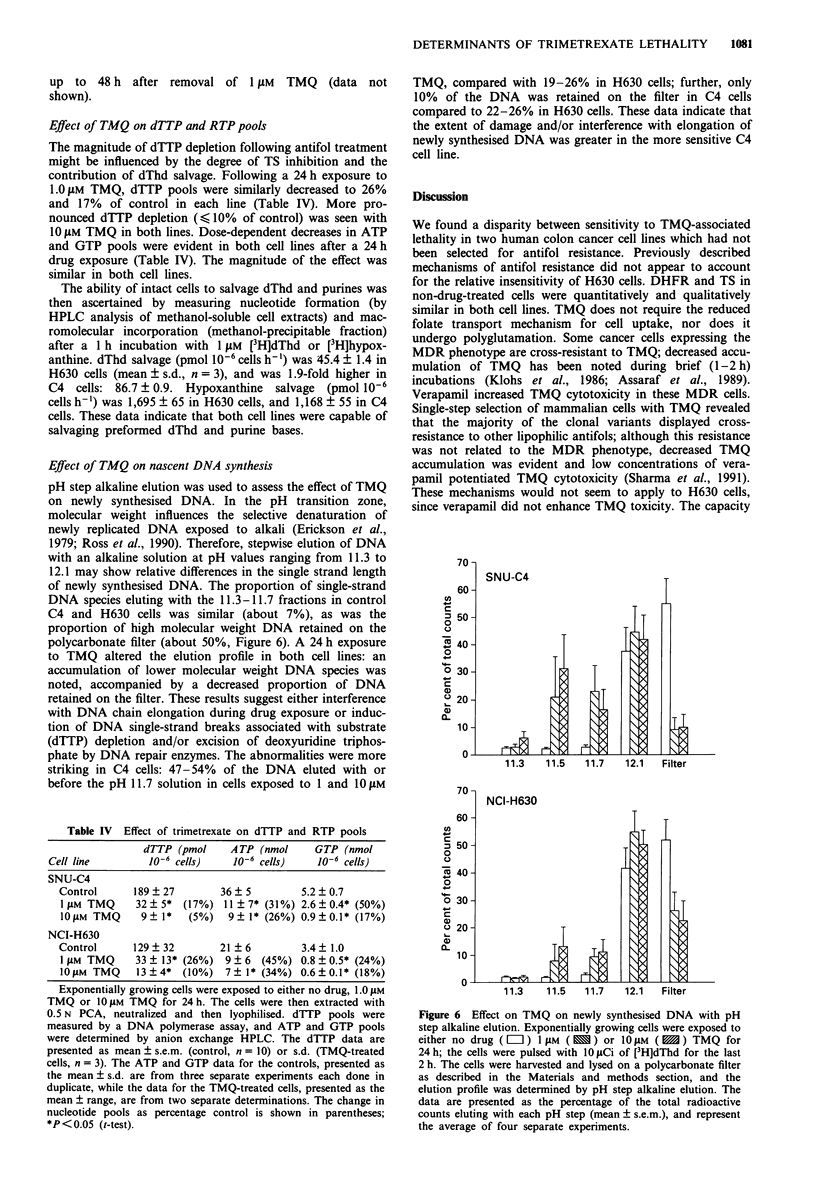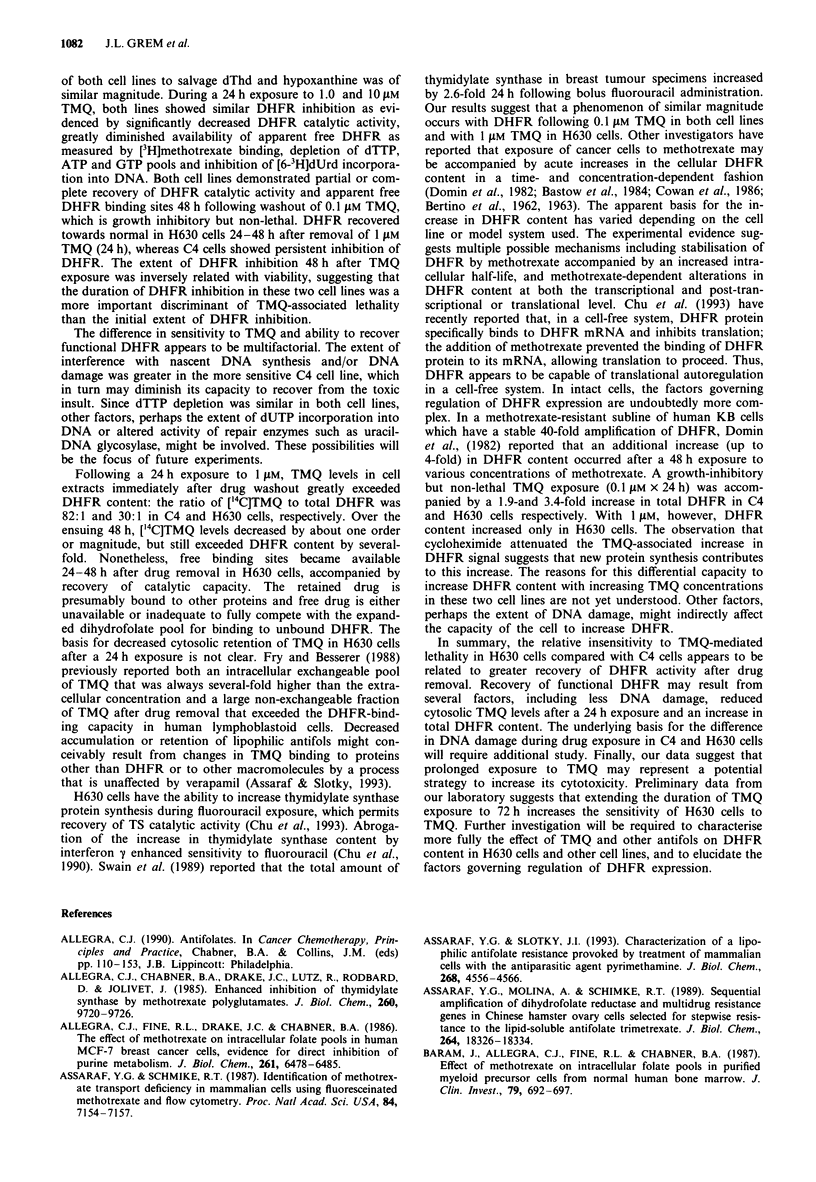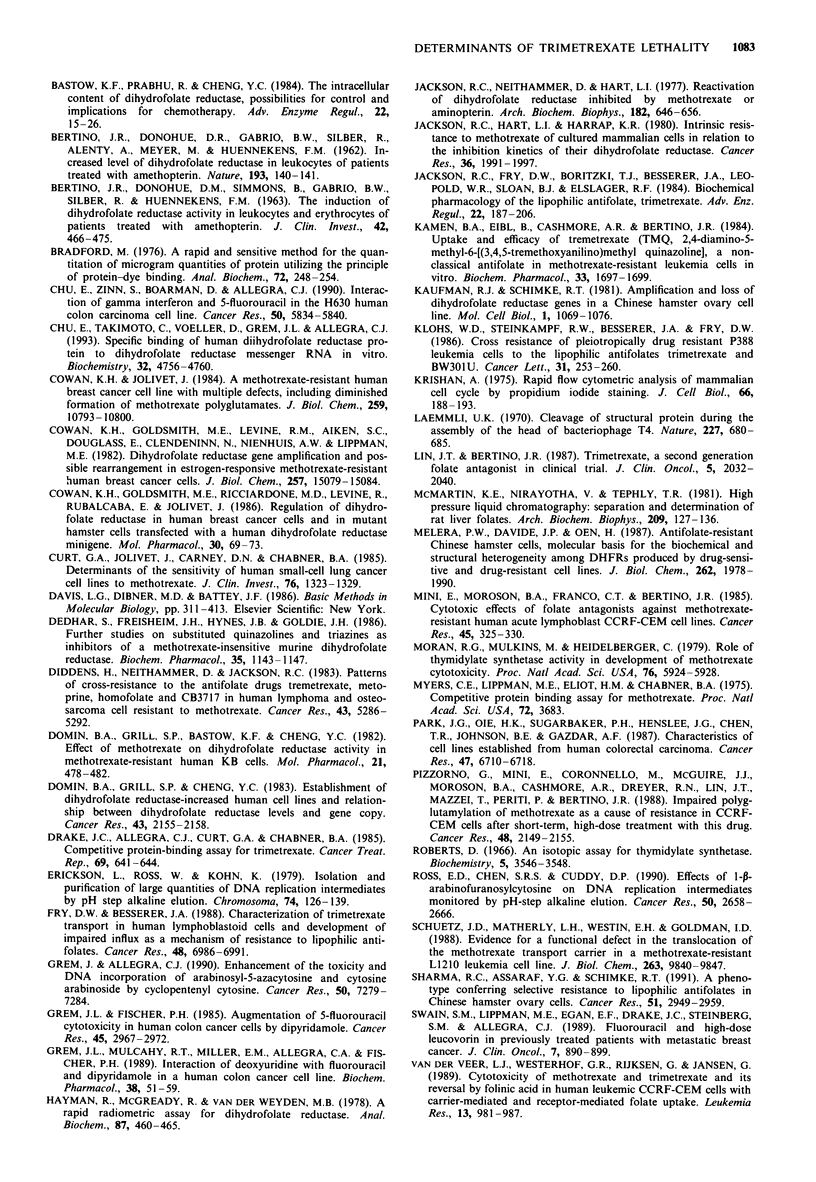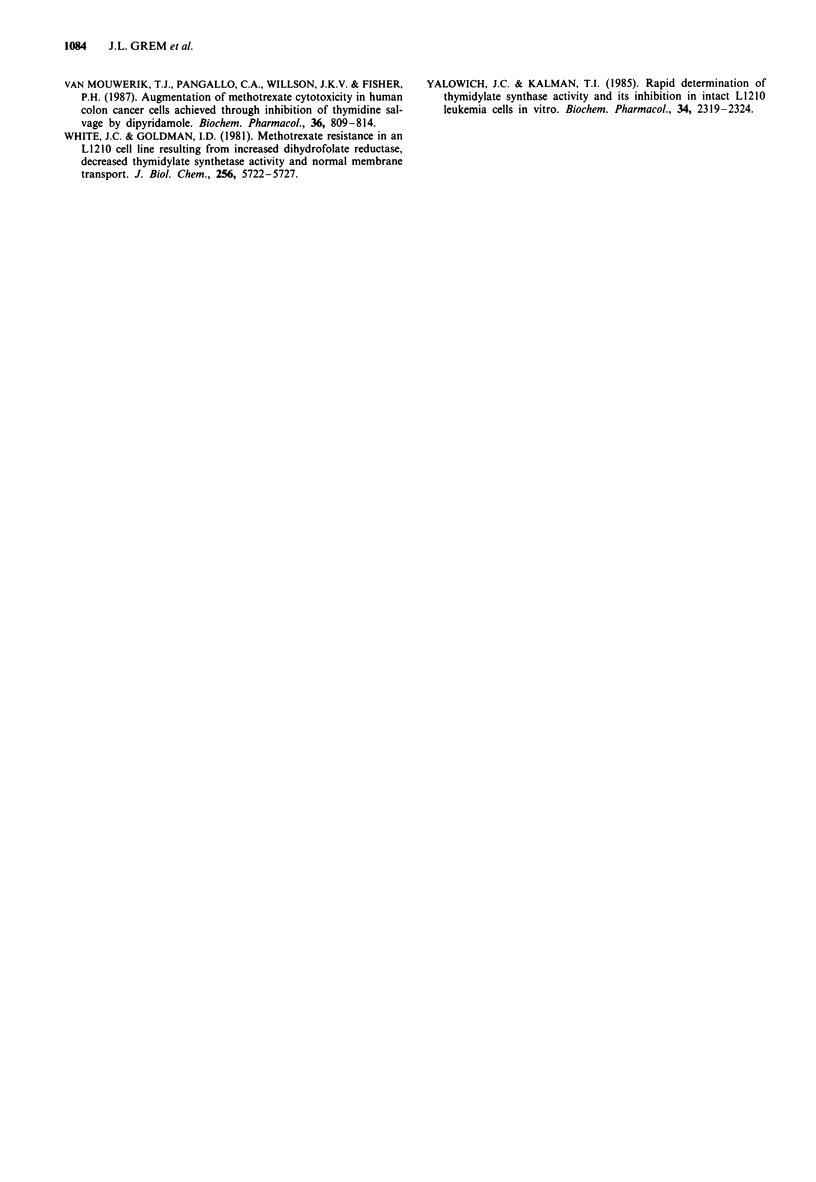# Determinants of trimetrexate lethality in human colon cancer cells.

**DOI:** 10.1038/bjc.1994.451

**Published:** 1994-12

**Authors:** J. L. Grem, D. M. Voeller, F. Geoffroy, E. Horak, P. G. Johnston, C. J. Allegra

**Affiliations:** National Cancer Institute-Navy Medical Oncology Branch, National Naval Medical Center, Bethesda, Maryland 20889-5105.

## Abstract

**Images:**


Br. J. Cancer (1994), 70, 1075–1084 © Macmillan Press Ltd., 1994

### Determinants of trimetrexate lethality in human colon cancer cells

J.L. Grem[1], D.M. Voeller[1], F. Geoffroy[1], E. Horak[2], P.G. Johnston[1] & C.J. Allegra[1]

[1]*National Cancer Institute-Navy Medical Oncology Branch, National Naval Medical Center, Building 8, Room 5101, Bethesda, Maryland 20889-5105, USA;* [2]*Laboratory of Tumor Virus Biology, Division of Cancer Etiology, National Cancer Institute, National Institutes of Health, Bethesda, Maryland 20892, USA.*

**Summary** We examined the cytotoxicity and biochemical effects of the lipophilic antifol trimetrexate (TMQ) in two human colon carcinoma cell lines, SNU-C4 and NCI-H630, with different inherent sensitivity to TMQ. While a 24 h exposure to 0.1 μM TMQ inhibited cell growth by 50–60% in both cell lines, it did not reduce clonogenic survival. A 24 h exposure to 1 and 10 μM TMQ produced 42% and 50% lethality in C4 cells, but did not affect H630 cells. Dihydrofolate reductase (DHFR) and thymidylate synthase were quantitatively and qualitatively similar in both lines. During drug exposure, DHFR catalytic activity was inhibited by ⩾ 85% in both cell lines; in addition, the reduction in apparent free DHFR binding capacity (⩽20% of control), depletion of dTTP, ATP and GTP pools and inhibition of [6-$^3$H]deoxyuridine incorporation into DNA were similar in C4 and H630 cells. TMQ produced a more striking alteration of the pH step alkaline elution profile of newly synthesised DNA in C4 cells compared with 630 cells, however, indicating greater interference with DNA chain elongation or more extensive DNA damage. When TMQ was removed after a 24 h exposure to 0.1 μM, recovery of DHFR catalytic activity and apparent free DHFR binding sites was evident over the next 24–48 h in both cell lines. With 1 and 10 μM, however, persistent inhibition of DHFR was evident in C4 cells, whereas DHFR recovered in H630 cells. These data suggest that, although DHFR inhibition during TMQ exposure produced growth inhibition, DHFR catalytic activity 48 h after drug removal was a more accurate predictor of lethality in these two cell lines. Several factors appeared to influence the duration of DHFR inhibition after drug removal, including initial TMQ concentration, declining cytosolic TMQ levels after drug removal, the ability to acutely increase total DHFR content and the extent of TMQ-mediated DNA damage. The greater sensitivity of C4 cells to TMQ-associated lethality may be attributed to the greater extent of TMQ-mediated DNA damage and more prolonged duration of DHFR inhibition after drug exposure.

Methotrexate, a 'classical' antifol, has been useful in the treatment of human neoplasms including acute lymphocytic leukaemia, non-Hodgkin's lymphoma, osteosarcoma, breast cancer and squamous cell carcinoma of the head and neck. Methotrexate is clinically inactive, however, against several human solid tumours, including colorectal carcinoma. Resistance to methotrexate has been attributed to numerous mechanisms (Allegra, 1990), including impaired membrane transport (Assaraf & Schimke, 1987; Schuetz *et al.*, 1988), defective polyglutamation (Cowan & Jolivet, 1984; Pizzorno *et al.*, 1988), reduced affinity of the target enzyme dihydrofolate reductase (EC 1.5.1.3, DHFR) (Jackson *et al.*, 1980; Melera *et al.*, 1987), increase in total DHFR activity due to gene amplification (Kaufman & Schimke, 1981; Cowan *et al.*, 1982; Domin *et al.*, 1983), decreased levels of thymidylate synthase (EC 2.1.1.45, TS) (White & Goldman, 1981; Curt *et al.*, 1985) and thymidine (dThd) salvage (Van Mouwerik *et al.*, 1987).

'Non-classical' antifols have been developed in an effort to overcome potential mechanisms of resistance. Trimetrexate (TMQ) is a 2,4-diaminoquinazoline antifol that is undergoing clinical evaluation as an antineoplastic agent and has been approved for the treatment of AIDS-related *Pneumocystis carinii* infection. Unlike methotrexate, TMQ is lipophilic and enters the cell by a transport process distinct from the reduced folate carrier (Diddens *et al.*, 1983; Jackson *et al.*, 1984; Kamen *et al.*, 1984). TMQ is a direct, potent inhibitor of DHFR, and does not undergo polyglutamation. In preclinical models, TMQ is active against several murine solid tumours which are refractory to methotrexate (Lin & Bertino, 1987). Cells which are resistant to methotrexate on the basis of impaired membrane transport are sensitive to TMQ (Mini *et al.*, 1985; Van Der Veer *et al.*, 1989). Multidrug-resistant (MDR) cell lines are cross-resistant to TMQ; verapamil enhances TMQ cytotoxicity in these cells (Klohs *et al.*, 1986; Assaraf *et al.*, 1989).

Despite the theoretical advantages of TMQ versus methotexate and its promising preclinical activity, clinical trials with TMQ in colorectal carcinoma have shown disappointing results. We therefore investigated the intrinsic determinants of sensitivity to TMQ in two human colorectal carcinoma cell lines with different sensitivity to TMQ-associated lethality. Such studies may suggest new strategies designed to overcome the intrinic resistance mechanisms to TMQ in colorectal cancer.

Correspondence: J.L. Grem.
Received 1 March 1994; and in revised form 29 July 1994.

### Materials and methods

TMQ, 6-*R*,*S*-5-formyltetrahydrofolate (leucovorin), and [$^{14}$C]-TMQ monoisethioate (sp. act. 13 mCi $mmol^{-1}$) were supplied by the Drug Synthesis and Chemistry Branch, Developmental Therapeutics Program, National Cancer Institute. The purity of the [$^{14}$C]TMQ was determined by high-performance liquid chromatography (HPLC); virtually 100% of the counts appeared in the fraction coeluting with TMQ standard. [3′,5′7-$^3$H]methotrexate (sp. act. 20 Ci $mmol^{-1}$), 6-*l*-[3′,5′,7-$^3$H]leucovorin (sp. act. 40 Ci $mmol^{-1}$), [6-$^3$H]dUrd (sp. act. 20 Ci $mmol^{-1}$), [3′,5′,7,9-$^3$H]folic acid (sp. act. 20 Ci $mmol^{-1}$) and [5-$^3$H]dUrd (sp. act. 15 Ci $mmol^{-1}$) were obtained from Moravek Biochemicals (Brea, CA, USA). The radiopurity of each compound as determined by HPLC was ⩾98%. Sep-pak $C_{18}$ cartridges and Pic Reagent A were purchased from Waters Chromatography Division of Millipore (Milford, MA, USA). Other chemicals were supplied by either Sigma (St. Louis, MO, USA), Aldrich Co. (Milwaukee, WI, USA) or J.T. Baker (Phillipsburg, NJ, USA). *Lactobacillus casei* TS (sp. act. = $5.4 \times 10^9$ mol $min^{-1}$ $mg^{-1}$) was from the New England Enzyme Center (Boston, MA, USA). 5,10-Methylene tetrahydrofolate was prepared as previously described (Grem *et al.*, 1989). Human DHFR purified from MCF-7 breast cancer cells was a generous gift from Dr Bernard Kaufman (National Institute of Diabetes and Digestive and Kidney Diseases, Bethesda, MD, USA).

### *Cell culture*

SNU-C4 and NCI-H630 cells are anchorage-dependent human colorectal cancer cell lines (Park *et al.*, 1987). Neither cell line has been selected for resistance to methotrexate or TMQ. The cells were grown in 'complete medium' consisting of RPMI-1640 medium supplemented with 7% non-dialysed fetal bovine serum and 1.8 mM glutamine (Biofluids, Rockville, MD, USA). Folate-free minimal essential medium was purchased from Gibco (Grand Island, NY, USA). Cell growth and colony formation were assessed as previously described (Grem & Fischer, 1985; Grem & Allegra, 1990). The amount ot tissue culture medium per $cm^2$ of tissue culture well or flask was constant for all experiments. For each type of experiment, the starting cell density was similar for both cell lines: 500 cells per 12.25 $cm^2$ for cloning experiments and 2,000–4,000 cells per $cm^2$ for cell growth and all other experiments.

*TMQ uptake*

Total cell-associated TMQ accumulation was determined according to the method of Kamen *et al.* (1984). Exponentially growing cells were exposed to [$^{14}$C]TMQ (4 µM) in folate-depleted medium. After incubation times of 5–60 min, the cells were washed three times with iced phosphate-buffered isotonic saline (PBS), and then were removed in 1 ml of distilled water by a cell scraper; residual radioactivity was quantitated in a liquid scintillation counter. Efflux was determined by incubating the cells with [$^{14}$C]TMQ for 30 min; the cells were then washed once with iced PBS and incubated in fresh folate-free medium. The cells were harvested at 5, 15, 30 and 60 min and the radioactivity was determined.

To determine intracellular accumulation and retention of TMQ, cells were exposed to 1 µM [$^{14}$C]TMQ in complete medium for 24 h. The cells were washed twice in RPMI and either harvested immediately or incubated in drug-free complete medium for an additional 24 or 48 h. To harvest, the cells were washed once with PBS, removed with a cell scraper, collected by centrifugation at 240 *g*, and frozen until analysis. The pellet was sonicated in 300 µl of 50 mM Tris–HCl (pH 7.4) containing 50 mM NADPH, and then centrifuged at 8,000 *g* for 45 min. The radioactivity in an aliquot of the lysate was determined. Protein concentrations were determined by the method of Bradford (1976).

*Assay of DHFR catalytic activity*

Exponentially growing cells were washed once with PBS, exposed to 20 mM EDTA (pH 7.5) for 20–30 s, then incubated in PBS at 37°C until the cells detached. The dislodged cells were washed once with ten volumes of iced PBS and collected by centrifugation at 800 *g* for 12 min. The cell pellet was then frozen until the day of assay. The cells were resuspended in 200–500 µl of 100 mM Tris–HCl buffer (pH 7.5), sonicated and the cytosol isolated by centrifugation as above. The catalytic activity of DHFR was quantitated using [$^3$H]dihydrofolate as the substrate (Dedhar *et al.*, 1986). [$^3$H]Dihydrofolate was synthesised by dithionite reduction of [$^3$H]dihydrofolate and purified by recrystalisation according to the method of Hayman *et al.* (1978). The reaction mixture, in a total volume of 200 µl, contained the following: 12.5 mM Tris–HCl pH 7.5, 0.2 M potassium chloride, 0.8 mM NADPH and 0.1 mM [$^3$H]dihydrofolate. The reaction was started by the addition of [$^3$H]dihydrofolate at 37°C. The reaction was quenched by placing the samples on ice and adding excess unlabelled folic acid (25 µl of 27 mM). Unreduced dihydrofolate and folic acid were precipitated by the addition of zinc sulphate (30 µl of 170 mM) and glacial acetic acid (10 µl). The sample was then centrifuged at 8,000 *g* for 30 min at 4°C; then the radioactivity in the supernatant minus the background was quantitated in a liquid scintillation counter. The background was determined from a duplicate sample to which no NADPH had been added. The reaction was linear for at least 20 min. The $IC_{50}$ of TMQ in the cytosolic assay was determined by preincubating the NADPH mixture with half-log increments of drug from 1 nM to 10 µM for 2 min at room temperature before adding [$^3$H]dihydrofolate.

To determine DHFR catalytic activity during and following TMQ exposure, cells were washed once with iced PBS, removed from the flask in iced PBS with a cell scraper and centrifuged as above. To maintain the stability of the TMQ bound to DHFR, the cell pellet was brought up in a total volume of 250 µl of 50 mM Tris (pH 7.4) containing 0.5 mM NADPH. Cytosol obtained from cells exposed to 500 µM TMQ for 1 h provided the background counts.

*Assay of DHFR-binding capacity*

The DHFR-binding assay was based on a modification of the protein binding assay described by Myers *et al.* (1975). Each 250 µl reaction mixture contained 100 mM Tris–HCl buffer (pH 7.5), various concentrations of [$^3$H]methotrexate, 0.22 mM NADPH and 0.2–0.5 mg of cytosolic protein. The contents of the tubes were mixed and allowed to equilibrate at room temperature for 10 min; 50 µl of a charcoal slurry (consisting of activated charcoal 10 g, bovine serum albumin 2.5 g, and 0.1 g of high molecular weight dextran in 100 ml of water) was then added. The samples were immediately mixed and centrifuged at 6,000 *g* for 30 min. A 150 µl aliquot of the supernatant was counted in a scintillation counter. Scatchard analysis was used to determine the dissociation constant ($K_d$).

Preparation of the cell extract and the binding assay itself were modified to permit quantitation of total DHFR binding capacity following TMQ exposure. Following a 24 h drug exposure, the medium was aspirated and the cells were washed three times with PBS. The cytosol was then obtained as described above and dialysed at 4°C against 4 l of 50 mM Tris–HCl (pH 8.5) per day using a prepared dialysis membrane (mol. wt. cut-off 6,000–8,000) and a microdialysis system (Bethesda Research Laboratories, Gaithersburg, MD, USA). The duration of dialysis was 24 h for 0.1 µM TMQ and 48 h for 1 µM TMQ. An aliquot of the cytosol was incubated with 300,000 d.p.m. [$^3$H]methotrexate and 100 mM Tris–HCl buffer (pH 7.5) for 3 h at 37°C. NADPH was then added (final concentration 0.22 mM) and the samples were allowed to equilibrate at room temperature for 10 min prior to the addition of an activated charcoal slurry. The samples were immediately vortexed and centrifuged at 6,000 *g* for 30 min; the radioactivity in an aliquot of the supernatant was determined.

The extraction procedure was modified further for separate experiments designed to measure the residual free DHFR binding sites following TMQ exposure. After a 24 h exposure to either diluent ($dH_2O$) or TMQ (0.1 and 1.0 µM), the medium was aspirated and the cells were washed once with iced PBS. The cells were harvested at 48 and 72 h (24 and 48 h after removal of TMQ) as described above. The cell pellet was resuspended in 250 µl of 100 mM Tris–HCl (pH 7.5) with NADPH (0.5 mM); after sonication, the lysate was collected after centrifugation. Additional NADPH (final concentration 1 mM) was directly added to the reaction mixture containing cell lysate, 100,000 d.p.m. [$^3$H]methotrexate, 100 mM Tris–HCl buffer, and the assay proceeded as previously outlined.

*Measurement of [$^3$H]methotrexate dissociation rate*

The dissociation rate of [$^3$H]methotrexate from DHFR in a cell-free assay was determined by a modification of the radioisotopic method described by Jackson *et al.* (1977). Briefly, 50,000 d.p.m. [$^3$H]methotrexate (20 µCi $nmol^{-1}$) was added to a 5-fold excess of DHFR in the presence of 0.1 µmol of NADPH and a physiological buffer (50 mM potassium phosphate pH 7.0, 50 mM sodium chloride) in a total reaction volume of 1 ml. The reaction was started by adding a 100-fold molar excess of unlabelled TMQ. At various intervals thereafter, 80 µl samples were taken and free [$^3$H]methotrexate was separated from that bound to DHFR by adsorption with 20 µl of a dextran–albumin-coated charcoal slurry and rapid filtration through a Gelman 0.45 µm Acrodisc (Potomac Scientific, Rockville, MD, USA) (Drake *et al.*, 1985). Aliquots of the effluent were counted, permitting measurement of the loss of bound [$^3$H]methotrexate from DHFR over time.

*Thymidylate synthase activity and binding*

TS activity in an aliquot of cytosol was assayed by a modification of a [5-$^3$H]dUMP release assay (Yalowich & Kalman, 1985; Roberts, 1966). dUMP pools were determined with the same assay except that excess exogenous TS and reduced folate were added, and the amount of dUMP was the limiting substrate (Grem *et al.*, 1989). The formation of a ternary complex consisting of TS, 5,10-methylene tetrahydrofolate and [6-$^3$H]5-FdUMP was studied using a binding assay (Moran *et al.*, 1979; Allegra *et al.*, 1986).

*Measurement of deoxy- and ribonucleotide triphosphate pools*

dTTP pools were monitored using a previously described enzymatic assay (Grem & Fischer, 1985). ATP and GTP pools were determined by anion-exchange HPLC as previously described (Grem & Allegra, 1990).

*Intracellular folate pool measurements*

Exponentially growing cells in folate-free medium supplemented with 0.1 µM *d,l*-leucovorin were exposed to [$^3$H]-leucovorin for a 72 h period, following which the cells were washed twice with PBS. Fresh medium was replaced, and then TMQ (1 µM) or $dH_2O$ was added for a 24 h period. The cells were either harvested immediately or washed twice with PBS and incubated in drug-free medium for an additional 24 h period. Folates were extracted, enzymatically hydrolysed to monoglutamate forms, then separated and quantitated by reversed-phase HPLC as previously described (McMartin *et al.*, 1981; Allegra *et al.*, 1986; Baram *et al.*, 1987).

*pH step alkaline elution of nascent DNA*

Exponentially growing cells were exposed to either no drug or 1 and 10 µM TMQ for 24 h; [$^3$H]dThd (10 µCi) was added during the final 2 h of exposure. The cells were then washed three times with ice-cold PBS, and the cells were detached by incubation with 20 mM disodium EDTA (pH 7.0). An equal number of cells was deposited on Nucleopore filters (25 mm, 1 µm pore size; Costar, Cambridge, MA, USA), held in an alkaline elution funnel (Millipore), then lysed in the dark with 5 ml of buffer containing 2 M sodium chloride, 0.3% sarcosyl detergent (pH 7.0) and 20 mM dosodium EDTA, pH 10.0 (Erickson *et al.*, 1979; Ross *et al.*, 1990). The lysed cells were washed with 5 ml of 20 mM disodium EDTA (pH 10) and then the exit tubing from the filter funnel was connected to a peristaltic pump (Minipuls III; Gilson Instrument, Middleton, WI, USA). A solution containing 20 mM EDTA (free acid form) adjusted to pH 11.3 with 1 M tetrapropylammoniumhydroxide (RSA, Ardsley, NY, USA) was added to the funnel and pumped through the filter at a rate of 0.08 ml min$^{-1}$. After 1 h, the solution was changed to one at pH 11.5, and the elution was continued for another hour. Fractions were collected with an automated fraction collector. This procedure was repeated for successive elutions at pH 11.7 and 12.1. The filter and elution fractions were neutralised with 150 µl of glacial acetic acid; scintillation fluid was added, and after a 24 h equilibration period the radioactivity was determined. The data were corrected for background counts.

*Cell cycle analysis*

The method of Krishan (1975) was used to evaluate the fraction of cells in each DNA cycle. Briefly, exponentially growing cells were exposed to no drug or to 1 µM TMQ for 24 h, then were lysed in a hypotonic solution in dilute citric acid. The nuclei were isolated, treated with DNAse-free RNAse, stained with propidium iodide, and then passed through a 35 µm nylon mesh. The cells were analysed on a Beckton-Dickinson flow cytometer FACScan using CellFIT software. The data were evaluated with the sum of broadened rectangles model.

*Western immunoblot analysis of DHFR content*

Equal amounts of cytosolic protein (200 µg) from either control or TMQ-treated cells were resolved by sodium dodecyl sulphate polyacrylamide gel electrophoresis using 15% acrylamide according to the method of Laemmli (1970). The gel was electroblotted onto a nitrocellulose membrane using a BioRad Transblot Semi-dry transfer cell with a Pharmacia electrophoresis power supply set at 200 mA for 2 h. Antibody staining was performed as described by Davis *et al.* (1986) with a human DHFR polyclonal antiserum (1:1000 dilution in Blotto buffer) that was obtained after serial i.v. injections (150 µg) of human DHFR purified from MCF-7 human breast cancer cells. A horseradish peroxidase-conjugated antibody (1:1,000 dilution in Blotto buffer) was used as the secondary antibody. Positive images were digitised by a ScanJet Plus scanner (Hewlett Packard) and analysed by NIH Image 1.36 software (Wayne Rasband, National Institute of Mental Health, Bethesda, MD, USA).

### Results

*Comparison of TMQ-induced lethality*

The doubling times during log-phase growth were similar: 18 h (C4 cells) and 22 h (H630 cells). After a 24 h exposure to TMQ followed by a 48 h drug-free period, the $IC_{50}$ values for growth inhibition were 0.05 (C4) and 0.1 µM (H630), respectively. A 24 h exposure to 0.1 µM TMQ, however, decreased colony formation by only 20% in C4 cells and had minimal effect in H630 cells (Figure 1). A 24 h exposure to 1 µM and 10 µM TMQ reduced cell growth by 78% ± 4% and 84% ± 3% of control in C4 cells, and clonogenic capacity was decreased by 42% and 50% respectively. In

**Figure 1** Effect of TMQ on clonogenic capacity. Exponentially growing cells (500) were replicately plated in six-well plates. The next day, varying concentrations of TMQ were added. Following a 24 h exposure, the cells were gently washed twice and fresh drug-free medium was replaced. Six days later, the cells were stained with methylene blue (0.25%) in 50% methanol, and colonies were enumerated. The data, presented as the mean ± s.e.m. are from ⩾4 separate experiments each done in duplicate: ▽, C4; ■, H630. The cloning efficiency of control cells was as follows: C4, 51% ± 5%; H630, 31% ± 2%. Similar results were obtained if a 2 day equilibrium period was allowed between plating and initial drug exposure.

H630 cells, a 24 h exposure to 1 and 10 μM TMQ reduced cell growth by 66% ± 6% and 72% ± 4% respectively, whereas colony formation was minimally affected. Concurrent exposure to 1 and 10 μM verapamil did not affect TMQ toxicity in either C4 or H630 cells. A 24 h exposure to TMQ in medium supplemented with dialysed FBS increased the toxicity; however, the H630 cells remained less sensitive than C4 cells. For example, with 1 μM TMQ, the colony number was reduced by 92% in C4 cells but only by 38% in H630 cells; 10 μM TMQ reduced colony formation in H630 cells by 70%.

### *TMQ uptake and retention*

During a 60 min exposure to 4 μM [$^{14}$C]TMQ in a folate-depleted medium, total cellular accumulation of TMQ occurred rapidly. An apparent plateau was reached by 30 min, and was similar in both cell lines (mean ± s.e.m., $n = 4$): C4, 276 ± 32 pmol $10^{-6}$ cells; H630, 250 ± 27 pmol $10^{-6}$ cells. Following removal of [$^{14}$C]TMQ, efflux occurred rapidly, and reached a plateau by 30 min, at which time the levels of cell-associated TMQ were similar in both lines (mean ± range: $n = 2$): C4, 30 ± 2 pmol $10^{-6}$ cells; H630, 32 ± 9 pmol $10^{-6}$ cells.

Because of the possibility that differences in TMQ accumulation might occur following a more prolonged exposure, [$^{14}$C]TMQ retention was also determined following a 24 h exposure (1 μM) in complete medium. Immediately after drug removal, cytosolic TMQ was 2.5-fold higher in C4 than in H630 cells (mean ± s.e.m. $n = 8$): 149 ± 8 *vs* 60 ± 4 pmol $mg^{-1}$ protein ($P<0.001$, paired *t*-test). TMQ levels decreased over the next 24 h in both cell lines, but remained 1.6-fold higher in C4 cells: 13.4 ± 1.4 *vs* 8.6 ± 0.7 pmol $mg^{-1}$ ($P = 0.003$). TMQ levels decreased only slightly thereafter (72 h, 9.2 ± 1.7 *vs* 8.5 ± 0.8 pmol $mg^{-1}$). Differences in TMQ retention after a 24 h exposure might therefore contribute to the disparity in sensitivity between the C4 and H630 cells.

### *Activity of dihydrofolate reductase and thymidylate synthase*

The characteristics of DHFR in cell-free assays from non-drug treated cells are shown in Table I. The [$^3$H]methotrexate-binding capacity of DHFR, which provides an index of DHFR content, was similar in the two cell lines. DHFR catalytic activity was also similar. DHFR from both cell lines was equally sensitive to TMQ-associated inhibition. The specific activity of [$^{14}$C]TMQ (13 mCi $mmol^{-1}$) was too low to permit its use in Scatchard analysis experiments. The calculated $K_D$ values determined with [$^3$H]methotrexate, however, were not significantly different. The ability of cold TMQ to displace [$^3$H]methotrexate from DHFR (the 'off-rate') was similar for the two cell lines. TS content and TS catalytic activity were not significantly different in these two lines.

**Table I** Characteristics of dihydrofolate reductase and thymidylate synthase

| | *SNU-C4* | *NCI-H630* |
|---|---|---|
| *DHFR* | | |
| Binding (pmol $mg^{-1}$) | 1.5 ± 0.2 | 1.0 ± 0.1 |
| Catalytic activity (pmol $min^{-1}$ $mg^{-1}$) | | |
| [$^3$H]Dihydrofolate assay | 1629 ± 250 | 1583 ± 176 |
| Turnover ($min^{-1}$) | 1058 | 1599 |
| $IC_{50}$ TMQ (nM) | 28 ± 12 | 32 ± 10 |
| $K_D$ (Scatchard analysis) | 204 ± 75 | 331 ± 116 |
| [$^3$H]Methotrexate off-rate (min) | 19 | 17 |
| *TS* | | |
| Binding (pmol $mg^{-1}$) | 42 ± 3 | 36 ± 8 |
| Catalytic activity (nmol $min^{-1}$ $mg^{-1}$) | 11.0 ± 1.3 | 6.9 ± 0.9 |
| Turnover ($min^{-1}$) | 262 | 192 |

The characteristics of DHFR and TS were determined as referenced in the methods section. The DHFR data, presented as the mean ± s.e.m., are from multiple separate experiments each assayed in duplicate (C4 and H630 respectively): binding, $n = 25$ and 28; catalytic, $n = 18$ and 23; Scatchard, $n = 6$ and 7; $IC_{50}$ TMQ, $n = 7$ and 6; the [$^3$H]methotrexate off-rate data are from one experiment done in triplicate (ratio of unlabelled TMQ to methotrexate = 100:1). The TS data, presented as the mean ± s.e.m. are from 5–6 separate experiments each run in duplicate.

### *Inhibition of DHFR during TMQ exposure*

Immediately following a 24 h exposure to 0.1 μM TMQ, apparent free DHFR binding capacity was decreased in both cell lines (Figure 2): C4, 0.39 pmol $mg^{-1}$ (27% of control); H630, 0.28 pmol $mg^{-1}$ (25% of control). Greater effects were seen with 1 μM TMQ for 24 h: C4, 0.16 pmol $mg^{-1}$ (11% of control); H630, 0.10 pmol $mg^{-1}$ (9% of control). The apparent values of unbound DHFR in this cell-free assay may overestimate the free binding sites *in situ* as a result of displacement of TMQ from DHFR during processing. While some displacement of TMQ during cell processing is unavoidable, we attempted to minimise this problem. Cells from both lines were harvested quickly in a uniform manner with minimal washing. NADPH was added to the cell pellet prior to sonication to promote stability of the TMQ–DHFR complex. The cell pellets were reconstituted with a uniform volume of buffer; finally, the cytosol was allowed to equilibrate with [$^3$H]methotrexate for only 10 min before the addition of charcoal to remove unbound [$^3$H]methotrexate.

Complementary studies revealed that DHFR catalytic activity was reduced to 26% and 46% of control in C4 and H630 cells, respectively, after a 24 h exposure to 0.1 μM TMQ (Figure 3). Exposure to higher concentrations of TMQ (⩾1 μM) resulted in greater inhibition of DHFR catalytic activity to ⩽15% of control in both cell lines. Thus, similar inhibition of DHFR was achieved during TMQ exposure in both cell lines as reflected by both the degree of occupied binding sites and greatly diminished catalytic activity.

### *Recovery of catalytically active DHFR following TMQ exposure*

Although DHFR inhibition in C4 and H630 cells was similar during drug exposure, we wished to test the possibility that the duration of DHFR inhibition might be different after drug removal. The apparent free DHFR binding sites in-

**Figure 2** Recovery of apparent free DHFR binding sites following TMQ exposure. C4 (▽, ▼) and H630 (O, ■) cells were exposed to 0.1 μM (open symbols) or 1.0 μM TMQ (closed symbols) for 24 h. The cells were harvested either immediately or 24 h and 48 h following drug removal. The cytosol was then isolated and the amount of apparent unbound DHFR binding sites was estimated by a competitive binding assay. The data, presented as pmol of [$^3$H]methotrexate bound per mg protein (mean ± s.e.m.) are from 4–11 separate experiments each run in duplicate.

creased 1.7-fold in C4 cells and 3.9-fold in H630 cells (to 46% and 100% of control, respectively, 48 h after removal of 0.1 μM TMQ; Figure 2). Following 1.0 μM TMQ, however, the apparent free DHFR binding sites increased by only 1.4-fold and 2.7-fold in C4 cells at 48 h and 72 h to 16% and 30% of control respectively. In H630 cells, in contrast, the apparent free DHFR binding capacity increased by 4.2-fold and 10.4-fold at 48 h and 72 h to 38% and 95% of control respectively. Thus, recovery of free DHFR binding sites appeared to be essentially complete 48 h following removal of 0.1 μM and 1 μM TMQ in the H630 cells.

Partial to complete recovery of DHFR catalytic activity was evident in both lines 48 h after removal of 0.1 μM TMQ (Figure 3). With ⩾1 μM TMQ, persistent inhibition of DHFR was evident in C4 cells for up to 48 h post washout of TMQ. Further, DHFR remained inhibited after 100 μM TMQ (catalytic activity <10% of control), a concentration associated with complete lethality. In contrast, H630 cells had essentially complete recovery of DHFR catalytic activity 48 h following 1 μM and 10 μM TMQ (to 96% and 75% of control respectively). With 100 and 500 μM TMQ, however, persistent inhibition of DHFR catalytic activity (⩽18% of control) was noted. The results suggest that, although DHFR

**Figure 3** Recovery of DHFR catalytic activity following TMQ exposure. Exponentially growing cells were exposed to various concentrations of TMQ for 24 h (○, 0.1 μM; ▽, 1 μM; □, 10 μM; ◇, 100 μM; △, 500 μM) then were either harvested immediately or gently washed and incubated in drug-free medium. The cell pellets were frozen until analysis; the pellets were the resuspended in 50 mM Tris-HCl buffer (pH 7.4) containing 0.5 mM NADPH, and the cytosol was isolated by centrifugation after sonication. DHFR catalytic activity was determined by measuring the concersion of [$^3$H]dihydrofolate to [$^3$H]reduced folates as described in Materials and methods. The data for drug-treated cells are shown as the percentage control DHFR catalytic activity (mean ± s.e.m.), and are from 4–8 separate determinations at each TMQ concentration: the activity in control cells was as follows (pmol min$^{-1}$ mg$^{-1}$): C4, 1,629 ± 250; H630, 1,583 ± 170.

inhibition during drug exposure was accompanied by growth inhibition, it did not closely correlate with lethality. In contrast, DHFR catalytic activity 48 h after TMQ exposure appeared to be a more accurate predictor for lethality in C4 and H630 cells (Figure 4).

We wished to verify the duration of DHFR inhibition in intact cells. Pulse incorporation of [6-$^3$H]dUrd into acid precipitable material provides an index of TS activity, and is affected by the availability of 5,10-methylenetrahydrofolate through the DHFR pathway. Since exposure to TMQ may result in dUMP accumulation, we measured the total endogenous dUMP pools following TMQ exposure to correct for dilution of [$^3$H]dUMP. Baseline dUMP pools were 1.9-fold higher in C4 cells (1513 ± 318 *vs* 813 ± 127 pmol $10^{-6}$ cells in H630 cells). Following 0.1 μM TMQ, dUMP pools were elevated 1.4-to 4.5-fold over baseline, and returned to baseline by 72 h (48 h following drug removal) in both cell lines. With 1.0 μM TMQ, however, persistent elevation of dUMP was noted for up to 72 h in both cell lines; the absolute dUMP levels were about 2-fold higher in C4 cells at each time point.

A 24 h exposure to 0.1 μM TMQ decreased [$^3$H]dUrd incorporation to 44% and 17% of control in H630 and C4 cells respectively (Table II). Immediately following a 24 h exposure to 1.0 μM TMQ, more pronounced inhibition was seen in both cell lines. In C4 cells, [$^3$H]dUrd incorporation remained ⩽20% of control at 48 h and 72 h (24 and 48 h following TMQ removal), while partial recovery occurred in H630 cells (H630 *vs* C4 at 48 h, $P = 0.02$).

As another indirect measure of DHFR activity, we determined the effect of a 24 h exposure to 1.0 μM TMQ on the intracellular folate pools in cells prelabelled with [$^3$H]leucovorin. Immediately following a 24 h TMQ exposure, [$^3$H]dihydrofolate was detected in both C4 and H630 cells, whereas none was detected in control cells (Table III). Following washout of TMQ and [$^3$H]leucovorin, the tritium in the total intracellular folate pool decreased over time, presumably as a result of exchange and dilutional effects resulting from continued cellular metabolism and efflux. The percent decrease in $^3$H in the total intracellular folate pool serves as an index of the decrement expected from these effects. In both cell lines, the total radiolabelled intracellular folate pool decreased by 38–50% over the ensuing 24 h period in both control and TMQ-treated cells. In C4 cells, the [$^3$H]dihydrofolate pool decreased by essentially the same proportion (55%), consistent with continued inhibition of

**Figure 4** Relationship of DHFR catalytic activity 48 h post removal of TMQ and clonogenic survival. DHFR catalytic activity 48 h following removal of TMQ as a percentage of control is plotted against the viability (per cent control); ■, H630; ▽, C4. The $r^2$ value for the linear regression was 0.91.

**Table II** Effect of TMQ on [$^3$H]dUrd incorporation into DNA

| *Condition* | *% control [$^3$H]dUrd incorporation* SNU-C4 (%) | NCI-H630 (%) |
|---|---|---|
| 0.1 μM TMQ | | |
| 24 h | 17 ± 5 | 43 ± 4 |
| 48 h (24 h post drug removal) | 31 ± 23 | 118 ± 45 |
| 72 h (48 h post drug removal) | 35 ± 14 | 67 ± 14 |
| 1.0 μM TMQ | | |
| 24 h | 10 ± 4 | 13 ± 7 |
| 48 h (24 h post drug removal) | 10 ± 7 | 60 ± 22 |
| 72 h (48 h post drug removal) | 18 ± 6 | 48 ± 9 |

Exponentially growing cells were exposed to 0.1 μM or 1.0 μM TMQ for 24 h, following which the cells were either harvested or gently washed free of drug and placed in drug-free medium. At the desired interval, cells were pulse labelled with [6-$^3$H]dUrd and the incorporation into acid-precipitable material was determined. The data, shown as mean ± s.e.m. are from 4–8 separate experiments each done in duplicate. The endogenous dUMP levels in control and TMQ-treated cells were determined ($n \geqslant 4$ experiments) with a modified tritium-release assay using [5-$^3$H]dUMP, and were then used to correct for differences in cold dUMP pools in drug-treated cells (data not shown). dUMP pools in control cells were as follows (pmol $10^{-6}$ cells, mean ± s.e.m.): C4, 1513 ± 318; H630, 813 ± 127.

**Table III** Effect of TMQ on [$^3$H[dihydrofolate metabolism in intact cells

| *Cell line* | *Condition* | *Dihydrofolate* | *Total intracellular [$^3$H]folate pool* |
|---|---|---|---|
| SNU-C4 | Control | | |
| | 24 h | 0 | 17.9 ± 4.8 |
| | 48 h | 0 | 10.5 ± 3.1 (59%) |
| | 1 μM TMQ | | |
| | 24 h | 2.2 ± 1.0 | 22.4 ± 5.9 |
| | 48 h | 1.0 ± 0.3 (45%) | 11.2 ± 4.7 (50%) |
| NCI-H630 | Control | | |
| | 24 h | 0 | 18.9 ± 7.4 |
| | 48 h | 0 | 11.8 ± 3.9 (62%) |
| | 1 μM TMQ | | |
| | 24 h | 6.4 ± 4.0 | 23.2 ± 5.8 |
| | 48 h | 1.4 ± 1.5 (22%) | 14.6 ± 5.8 (63%) |

Exponentially growing cells in folate-free medium supplemented with 0.1 μM leucovorin were prelabelled with [$^3$H]leucovorin for 72 h, following which the cells were gently washed and then exposed to $dH_2O$ or 1.0 μM TMQ for 24 h. Either immediately after the drug exposure or 24 h following washout of TMQ, cells were extracted as described in the Materials and methods section. After enzymatic hydrolysis of the polyglutamated residues, the [$^3$H]folate pools were determined by reversed-phase HPLC. The data, presented as pmol folate per mg protein (mean ± s.d.), are from three separate experiments. The size of the folate pool at 48 h as a percentage of the folate pool at 24 h is shown in parentheses.

DHFR. In the H630 cells, in contrast, the decline in the [$^3$H]dihydrofolate pool, 78%, appeared to be greater than expected on the basis of exchange and dilutional effects, consistent with partial recovery of DHFR and resumption of dihydrofolate utilization.

### *Effect of TMQ exposure on total DHFR content*

To test the hypothesis that total DHFR content may change following a 24 h TMQ exposure, cytosol obtained from control and drug-treated cells was extensively dialysed to remove free TMQ and drug bound to DHFR. A longer (3 h) incubation with [$^3$H]methotrexate was also used to permit complete exchange with any residual bound TMQ. The adequacy of dialysis was assessed by comparing the DHFR binding capacity of cells harvested immediately after 1 h exposure to TMQ with control values. We reasoned that a 1 h exposure would be too brief to result in changes in total levels of DHFR; therefore, if DHFR levels measured after a 1 h drug exposure were similar to control levels, the dialysis was assumed to be complete. After a 24 h and 48 h period of dialysis, the DHFR binding capacity in cytosols from cells exposed for 1 h to 0.1 and 1.0 μM TMQ, respectively, was 1.2-fold (± 0.1) of control values in both cell lines, indicating that essentially all TMQ had been removed from DHFR. Immediately after a 24 h exposure to 0.1 μM TMQ, however, an increase in total DHFR binding over baseline was evident in both lines (pmol $mg^{-1}$): C4, 3.3 ± 0.6 ($n = 6$) *vs* 1.7 ± 0.2 ($n = 16$, $P = 0.004$); H630, 3.1 ± 0.4 ($n = 9$) *vs* 0.9 ± 0.1 ($n = 14$, $P < 0.001$). With 1 μM TMQ, no increase in DHFR binding capacity was seen in C4 cells (1.8 ± 0.3 pmol $mg^{-1}$, $n = 8$). A 2.2-fold increase over baseline DHFR binding occurred, however, in H630 cells: 2.0 ± 0.2 pmol $mg^{-1}$ ($n = 6$, $P > 0.001$). With 10 μM TMQ, removal of bound drug was incomplete after 72 h of dialysis. Prolonged dialysis beyond 72 h resulted in progressive loss of enzymatic activity; therefore, experiments using higher concentrations could not be reliably interpreted using the binding assay.

Because DHFR content may vary in different phases of the cell cycle, it was important to ascertain the effect of TMQ on cell cycle distribution. The proportion of control cells in S-phase was similar in both cell lines (C4, 45% ± 17%; H630 cells, 48% ± 14%, mean ± s.d., $n = 3$). A 24 h exposure to 1 μM TMQ was associated with an 11–12% increase in the proportion of cells in S-phase in both cell lines (C4, 56% ± 36%; H630, 60% ± 24%). No change was seen in the proportion of cells in $G_0/G_1$. TMQ exposure was accompanied by a block in the entry of cells into $G_2/M$ phase (control *vs* TMQ-treated, mean ± s.d.): C4, 11% ± 2% *vs* 0.7% ± 0.7%; H630, 12% ± 1% *vs* 0.6% ± 0.1%. Thus, 1 μM TMQ produced comparable effects on the cell cycle in each cell line.

An increase in DHFR protein levels was confirmed by Western immunoblot analysis in H630 cells treated with 1 μM TMQ for 24 h (Figure 5), whereas no appreciable change was evident in C4 cells. To determine whether new protein synthesis contributed to this phenomenon, the effect of a 24 h exposure to cycloheximide (CHEX) on [$^{35}$S]methionine incorporation into acid-precipitable material was determined. Then, the effect of concurrent exposure to CHEX and TMQ was investigated. A 24 h exposure to CHEX with 1 μM TMQ attenuated the TMQ-associated increase in DHFR content in a dose-dependent fashion (fold increase in DHFR densitometry signal relative to control): TMQ alone, 2.5-fold; TMQ + 5 μM CHEX (which inhibited protein synthesis by 40%), 1.7-fold; TMQ + 50 μM CHEX (which inhibited protein synthesis by 79%), 1.2-fold. DHFR content in H630 cells remained 2.4- to 2.8-fold higher relative to control for

**Figure 5** Western immunoblot analysis of DHFR content following TMQ exposure in H630 cells. Equal amounts of cytosolic protein (200 μg) from control or TMQ-treated cells (1 μM for 24 h) were resolved by SDS-polyacrylamide gel electrophoresis (15% acrylamide). A human DHFR polyclonal antiserum with a horse radish peroxidase-conjugated antibody as the secondary antibody was used to detect the DHFR protein. The contents of the lanes are as follows (relative densitometry units are shown in parentheses): A, protein molecular weight markers; B, purified human DHFR; C and D, control (0.057 and 0.029 U); E and F, TMQ-treated cells (0.131 and 0.123 U).

up to 48 h after removal of 1 μM TMQ (data not shown).

*Effect of TMQ on dTTP and RTP pools*

The magnitude of dTTP depletion following antifol treatment might be influenced by the degree of TS inhibition and the contribution of dThd salvage. Following a 24 h exposure to 1.0 μM TMQ, dTTP pools were similarly decreased to 26% and 17% of control in each line (Table IV). More pronounced dTTP depletion ($\leqslant$10% of control) was seen with 10 μM TMQ in both lines. Dose-dependent decreases in ATP and GTP pools were evident in both cell lines after a 24 h drug exposure (Table IV). The magnitude of the effect was similar in both cell lines.

The ability of intact cells to salvage dThd and purines was then ascertained by measuring nucleotide formation (by HPLC analysis of methanol-soluble cell extracts) and macromolecular incorporation (methanol-precipitable fraction) after a 1 h incubation with 1 μM [$^3$H]dThd or [$^3$H]hypoxanthine. dThd salvage (pmol $10^{-6}$ cells $h^{-1}$) was 45.4 ± 1.4 in H630 cells (mean ± s.d., $n = 3$), and was 1.9-fold higher in C4 cells: 86.7 ± 0.9. Hypoxanthine salvage (pmol $10^{-6}$ cells $h^{-1}$) was 1,695 ± 65 in H630 cells, and 1,168 ± 55 in C4 cells. These data indicate that both cell lines were capable of salvaging preformed dThd and purine bases.

*Effect of TMQ on nascent DNA synthesis*

pH step alkaline elution was used to assess the effect of TMQ on newly synthesised DNA. In the pH transition zone, molecular weight influences the selective denaturation of newly replicated DNA exposed to alkali (Erickson *et al.*, 1979; Ross *et al.*, 1990). Therefore, stepwise elution of DNA with an alkaline solution at pH values ranging from 11.3 to 12.1 may show relative differences in the single strand length of newly synthesised DNA. The proportion of single-strand DNA species eluting with the 11.3–11.7 fractions in control C4 and H630 cells was similar (about 7%), as was the proportion of high molecular weight DNA retained on the polycarbonate filter (about 50%, Figure 6). A 24 h exposure to TMQ altered the elution profile in both cell lines: an accumulation of lower molecular weight DNA species was noted, accompanied by a decreased proportion of DNA retained on the filter. These results suggest either interference with DNA chain elongation during drug exposure or induction of DNA single-strand breaks associated with substrate (dTTP) depletion and/or excision of deoxyuridine triphosphate by DNA repair enzymes. The abnormalities were more striking in C4 cells: 47–54% of the DNA eluted with or before the pH 11.7 solution in cells exposed to 1 and 10 μM TMQ, compared with 19–26% in H630 cells; further, only 10% of the DNA was retained on the filter in C4 cells compared to 22–26% in H630 cells. These data indicate that the extent of damage and/or interference with elongation of newly synthesised DNA was greater in the more sensitive C4 cell line.

### Discussion

We found a disparity between sensitivity to TMQ-associated lethality in two human colon cancer cell lines which had not been selected for antifol resistance. Previously described mechanisms of antifol resistance did not appear to account for the relative insensitivity of H630 cells. DHFR and TS in non-drug-treated cells were quantitatively and qualitatively similar in both cell lines. TMQ does not require the reduced folate transport mechanism for cell uptake, nor does it undergo polyglutamation. Some cancer cells expressing the MDR phenotype are cross-resistant to TMQ; decreased accumulation of TMQ has been noted during brief (1–2 h) incubations (Klohs *et al.*, 1986; Assaraf *et al.*, 1989). Verapamil increased TMQ cytotoxicity in these MDR cells. Single-step selection of mammalian cells with TMQ revealed that the majority of the clonal variants displayed cross-resistance to other lipophilic antifols; although this resistance was not related to the MDR phenotype, decreased TMQ accumulation was evident and low concentrations of verapamil potentiated TMQ cytotoxicity (Sharma *et al.*, 1991). These mechanisms would not seem to apply to H630 cells, since verapamil did not enhance TMQ toxicity. The capacity

**Table IV** Effect of trimetrexate on dTTP and RTP pools

| Cell line | *dTTP (pmol $10^{-6}$ cells)* | | *ATP (nmol $10^{-6}$ cells)* | | *GTP (nmol $10^{-6}$ cells)* | |
|---|---|---|---|---|---|---|
| SNU-C4 | | | | | | |
| Control | 189 ± 27 | | 36 ± 5 | | 5.2 ± 0.7 | |
| 1 μM TMQ | 32 ± 5* | (17%) | 11 ± 7* | (31%) | 2.6 ± 0.4* | (50%) |
| 10 μM TMQ | 9 ± 1* | (5%) | 9 ± 1* | (26%) | 0.9 ± 0.1* | (17%) |
| NCI-H630 | | | | | | |
| Control | 129 ± 32 | | 21 ± 6 | | 3.4 ± 1.0 | |
| 1 μM TMQ | 33 ± 13* | (26%) | 9 ± 6 | (45%) | 0.8 ± 0.5* | (24%) |
| 10 μM TMQ | 13 ± 4* | (10%) | 7 ± 1* | (34%) | 0.6 ± 0.1* | (18%) |

Exponentially growing cells were exposed to either no drug, 1.0 μM TMQ or 10 μM TMQ for 24 h. The cells were then extracted with 0.5 N PCA, neutralized and then lyophilised. dTTP pools were measured by a DNA polymerase assay, and ATP and GTP pools were determined by anion exchange HPLC. The dTTP data are presented as mean ± s.e.m. (control, $n = 10$) or s.d. (TMQ-treated cells, $n = 3$). The ATP and GTP data for the controls, presented as the mean ± s.d. are from three separate experiments each done in duplicate, while the data for the TMQ-treated cells, presented as the mean ± range, are from two separate determinations. The change in nucleotide pools as percentage control is shown in parentheses; $*P < 0.05$ (*t*-test).

**Figure 6** Effect on TMQ on newly synthesised DNA with pH step alkaline elution. Exponentially growing cells were exposed to either no drug (□) 1 μM (▧) or 10 μM (▨) TMQ for 24 h; the cells were pulsed with 10 μCi of [$^3$H]dThd for the last 2 h. The cells were harvested and lysed on a polycarbonate filter as described in the Materials and methods section, and the elution profile was determined by pH step alkaline elution. The data are presented as the percentage of the total radioactive counts eluting with each pH step (mean ± s.e.m.), and represent the average of four separate experiments.

of both cell lines to salvage dThd and hypoxanthine was of similar magnitude. During a 24 h exposure to 1.0 and 10 μM TMQ, both lines showed similar DHFR inhibition as evidenced by significantly decreased DHFR catalytic activity, greatly diminished availability of apparent free DHFR as measured by [$^3$H]methotrexate binding, depletion of dTTP, ATP and GTP pools and inhibition of [6-$^3$H]dUrd incorporation into DNA. Both cell lines demonstrated partial or complete recovery of DHFR catalytic activity and apparent free DHFR binding sites 48 h following washout of 0.1 μM TMQ, which is growth inhibitory but non-lethal. DHFR recovered towards normal in H630 cells 24–48 h after removal of 1 μM TMQ (24 h), whereas C4 cells showed persistent inhibition of DHFR. The extent of DHFR inhibition 48 h after TMQ exposure was inversely related with viability, suggesting that the duration of DHFR inhibition in these two cell lines was a more important discriminant of TMQ-associated lethality than the initial extent of DHFR inhibition.

The difference in sensitivity to TMQ and ability to recover functional DHFR appears to be multifactorial. The extent of interference with nascent DNA synthesis and/or DNA damage was greater in the more sensitive C4 cell line, which in turn may diminish its capacity to recover from the toxic insult. Since dTTP depletion was similar in both cell lines, other factors, perhaps the extent of dUTP incorporation into DNA or altered activity of repair enzymes such as uracil-DNA glycosylase, might be involved. These possibilities will be the focus of future experiments.

Following a 24 h exposure to 1 μM, TMQ levels in cell extracts immediately after drug washout greatly exceeded DHFR content: the ratio of [$^{14}$C]TMQ to total DHFR was 82:1 and 30:1 in C4 and H630 cells, respectively. Over the ensuing 48 h, [$^{14}$C]TMQ levels decreased by about one order or magnitude, but still exceeded DHFR content by several-fold. Nonetheless, free binding sites became available 24–48 h after drug removal in H630 cells, accompanied by recovery of catalytic capacity. The retained drug is presumably bound to other proteins and free drug is either unavailable or inadequate to fully compete with the expanded dihydrofolate pool for binding to unbound DHFR. The basis for decreased cytosolic retention of TMQ in H630 cells after a 24 h exposure is not clear. Fry and Besserer (1988) previously reported both an intracellular exchangeable pool of TMQ that was always several-fold higher than the extracellular concentration and a large non-exchangeable fraction of TMQ after drug removal that exceeded the DHFR-binding capacity in human lymphoblastoid cells. Decreased accumulation or retention of lipophilic antifols might conceivably result from changes in TMQ binding to proteins other than DHFR or to other macromolecules by a process that is unaffected by verapamil (Assaraf & Slotky, 1993).

H630 cells have the ability to increase thymidylate synthase protein synthesis during fluorouracil exposure, which permits recovery of TS catalytic activity (Chu *et al.*, 1993). Abrogation of the increase in thymidylate synthase content by interferon γ enhanced sensitivity to fluorouracil (Chu *et al.*, 1990). Swain *et al.* (1989) reported that the total amount of thymidylate synthase in breast tumour specimens increased by 2.6-fold 24 h following bolus fluorouracil administration. Our results suggest that a phenomenon of similar magnitude occurs with DHFR following 0.1 μM TMQ in both cell lines and with 1 μM TMQ in H630 cells. Other investigators have reported that exposure of cancer cells to methotrexate may be accompanied by acute increases in the cellular DHFR content in a time- and concentration-dependent fashion (Domin *et al.*, 1982; Bastow *et al.*, 1984; Cowan *et al.*, 1986; Bertino *et al.*, 1962, 1963). The apparent basis for the increase in DHFR content has varied depending on the cell line or model system used. The experimental evidence suggests multiple possible mechanisms including stabilisation of DHFR by methotrexate accompanied by an increased intracellular half-life, and methotrexate-dependent alterations in DHFR content at both the transcriptional and post-transcriptional or translational level. Chu *et al.* (1993) have recently reported that, in a cell-free system, DHFR protein specifically binds to DHFR mRNA and inhibits translation; the addition of methotrexate prevented the binding of DHFR protein to its mRNA, allowing translation to proceed. Thus, DHFR appears to be capable of translational autoregulation in a cell-free system. In intact cells, the factors governing regulation of DHFR expression are undoubtedly more complex. In a methotrexate-resistant subline of human KB cells which have a stable 40-fold amplification of DHFR, Domin *et al.*, (1982) reported that an additional increase (up to 4-fold) in DHFR content occurred after a 48 h exposure to various concentrations of methotrexate. A growth-inhibitory but non-lethal TMQ exposure (0.1 μM × 24 h) was accompanied by a 1.9-and 3.4-fold increase in total DHFR in C4 and H630 cells respectively. With 1 μM, however, DHFR content increased only in H630 cells. The observation that cycloheximide attenuated the TMQ-associated increase in DHFR signal suggests that new protein synthesis contributes to this increase. The reasons for this differential capacity to increase DHFR content with increasing TMQ concentrations in these two cell lines are not yet understood. Other factors, perhaps the extent of DNA damage, might indirectly affect the capacity of the cell to increase DHFR.

In summary, the relative insensitivity to TMQ-mediated lethality in H630 cells compared with C4 cells appears to be related to greater recovery of DHFR activity after drug removal. Recovery of functional DHFR may result from several factors, including less DNA damage, reduced cytosolic TMQ levels after a 24 h exposure and an increase in total DHFR content. The underlying basis for the difference in DNA damage during drug exposure in C4 and H630 cells will require additional study. Finally, our data suggest that prolonged exposure to TMQ may represent a potential strategy to increase its cytotoxicity. Preliminary data from our laboratory suggests that extending the duration of TMQ exposure to 72 h increases the sensitivity of H630 cells to TMQ. Further investigation will be required to characterise more fully the effect of TMQ and other antifols on DHFR content in H630 cells and other cell lines, and to elucidate the factors governing regulation of DHFR expression.

### References

ALLEGRA, C.J. (1990). Antifolates. In *Cancer Chemotherapy, Principles and Practice*, Chabner, B.A. & Collins, J.M. (eds) pp. 110–153, J.B. Lippincott: Philadelphia.

ALLEGRA, C.J., CHABNER, B.A., DRAKE, J.C., LUTZ, R., RODBARD, D. & JOLIVET, J. (1985). Enhanced inhibition of thymidylate synthase by methotrexate polyglutamates. *J. Biol. Chem.*, **260**, 9720–9726.

ALLEGRA, C.J., FINE, R.L., DRAKE, J.C. & CHABNER, B.A. (1986). The effect of methotrexate on intracellular folate pools in human MCF-7 breast cancer cells, evidence for direct inhibition of purine metabolism. *J. Biol. Chem.*, **261**, 6478–6485.

ASSARAF, Y.G. & SCHMIKE, R.T. (1987). Identification of methotrexate transport deficiency in mammalian cells using fluoresceinated methotrexate and flow cytometry. *Proc. Natl Acad. Sci. USA*, **84**, 7154–7157.

ASSARAF, Y.G. & SLOTKY, J.I. (1993). Characterization of a lipophilic antifolate resistance provoked by treatment of mammalian cells with the antiparasitic agent pyrimethamine. *J. Biol. Chem.*, **268**, 4556–4566.

ASSARAF, Y.G., MOLINA, A. & SCHIMKE, R.T. (1989). Sequential amplification of dihydrofolate reductase and multidrug resistance genes in Chinese hamster ovary cells selected for stepwise resistance to the lipid-soluble antifolate trimetrexate. *J. Biol. Chem.*, **264**, 18326–18334.

BARAM, J., ALLEGRA, C.J., FINE, R.L. & CHABNER, B.A. (1987). Effect of methotrexate on intracellular folate pools in purified myeloid precursor cells from normal human bone marrow. *J. Clin. Invest.*, **79**, 692–697.

BASTOW, K.F., PRABHU, R. & CHENG, Y.C. (1984). The intracellular content of dihydrofolate reductase, possibilities for control and implications for chemotherapy. *Adv. Enzyme Regul.*, **22**, 15–26.

BERTINO, J.R., DONOHUE, D.R., GABRIO, B.W., SILBER, R., ALENTY, A., MEYER, M. & HUENNEKENS, F.M. (1962). Increased level of dihydrofolate reductase in leukocytes of patients treated with amethopterin. *Nature*, **193**, 140–141.

BERTINO, J.R., DONOHUE, D.M., SIMMONS, B., GABRIO, B.W., SILBER, R. & HUENNEKENS, F.M. (1963). The induction of dihydrofolate reductase activity in leukocytes and erythrocytes of patients treated with amethopterin. *J. Clin. Invest.*, **42**, 466–475.

BRADFORD, M. (1976). A rapid and sensitive method for the quantitation of microgram quantities of protein utilizing the principle of protein–dye binding. *Anal. Biochem.*, **72**, 248–254.

CHU, E., ZINN, S., BOARMAN, D. & ALLEGRA, C.J. (1990). Interaction of gamma interferon and 5-fluorouracil in the H630 human colon carcinoma cell line. *Cancer Res.*, **50**, 5834–5840.

CHU, E., TAKIMOTO, C., VOELLER, D., GREM, J.L. & ALLEGRA, C.J. (1993). Specific binding of human diihydrofolate reductase protein to dihydrofolate reductase messenger RNA in vitro. *Biochemistry*, **32**, 4756–4760.

COWAN, K.H. & JOLIVET, J. (1984). A methotrexate-resistant human breast cancer cell line with multiple defects, including diminished formation of methotrexate polyglutamates. *J. Biol. Chem.*, **259**, 10793–10800.

COWAN, K.H., GOLDSMITH, M.E., LEVINE, R.M., AIKEN, S.C., DOUGLASS, E., CLENDENINN, N., NIENHUIS, A.W. & LIPPMAN, M.E. (1982). Dihydrofolate reductase gene amplification and possible rearrangement in estrogen-responsive methotrexate-resistant human breast cancer cells. *J. Biol. Chem.*, **257**, 15079–15084.

COWAN, K.H., GOLDSMITH, M.E., RICCIARDONE, M.D., LEVINE, R., RUBALCABA, E. & JOLIVET, J. (1986). Regulation of dihydrofolate reductase in human breast cancer cells and in mutant hamster cells transfected with a human dihydrofolate reductase minigene. *Mol. Pharmacol.*, **30**, 69–73.

CURT, G.A., JOLIVET, J., CARNEY, D.N. & CHABNER, B.A. (1985). Determinants of the sensitivity of human small-cell lung cancer cell lines to methotrexate. *J. Clin. Invest.*, **76**, 1323–1329.

DAVIS, L.G., DIBNER, M.D. & BATTEY, J.F. (1986). *Basic Methods in Molecular Biology*, pp. 311–413. Elsevier Scientific: New York.

DEDHAR, S., FREISHEIM, J.H., HYNES, J.B. & GOLDIE, J.H. (1986). Further studies on substituted quinazolines and triazines as inhibitors of a methotrexate-insensitive murine dihydrofolate reductase. *Biochem. Pharmacol.*, **35**, 1143–1147.

DIDDENS, H., NEITHAMMER, D. & JACKSON, R.C. (1983). Patterns of cross-resistance to the antifolate drugs tremetrexate, metoprine, homofolate and CB3717 in human lymphoma and osteosarcoma cell resistant to methotrexate. *Cancer Res.*, **43**, 5286–5292.

DOMIN, B.A., GRILL, S.P., BASTOW, K.F. & CHENG, Y.C. (1982). Effect of methotrexate on dihydrofolate reductase activity in methotrexate-resistant human KB cells. *Mol. Pharmacol.*, **21**, 478–482.

DOMIN, B.A., GRILL, S.P. & CHENG, Y.C. (1983). Establishment of dihydrofolate reductase-increased human cell lines and relationship between dihydrofolate reductase levels and gene copy. *Cancer Res.*, **43**, 2155–2158.

DRAKE, J.C., ALLEGRA, C.J., CURT, G.A. & CHABNER, B.A. (1985). Competitive protein-binding assay for trimetrexate. *Cancer Treat. Rep.*, **69**, 641–644.

ERICKSON, L., ROSS, W. & KOHN, K. (1979). Isolation and purification of large quantities of DNA replication intermediates by pH step alkaline elution. *Chromosoma*, **74**, 126–139.

FRY, D.W. & BESSERER, J.A. (1988). Characterization of trimetrexate transport in human lymphoblastoid cells and development of impaired influx as a mechanism of resistance to lipophilic antifolates. *Cancer Res.*, **48**, 6986–6991.

GREM, J. & ALLEGRA, C.J. (1990). Enhancement of the toxicity and DNA incorporation of arabinosyl-5-azacytosine and cytosine arabinoside by cyclopentenyl cytosine. *Cancer Res.*, **50**, 7279–7284.

GREM, J.L. & FISCHER, P.H. (1985). Augmentation of 5-fluorouracil cytotoxicity in human colon cancer cells by dipyridamole. *Cancer Res.*, **45**, 2967–2972.

GREM, J.L., MULCAHY, R.T., MILLER, E.M., ALLEGRA, C.A. & FISCHER, P.H. (1989). Interaction of deoxyuridine with fluorouracil and dipyridamole in a human colon cancer cell line. *Biochem. Pharmacol.*, **38**, 51–59.

HAYMAN, R., MCGREADY, R. & VAN DER WEYDEN, M.B. (1978). A rapid radiometric assay for dihydrofolate reductase. *Anal. Biochem.*, **87**, 460–465.

JACKSON, R.C., NEITHAMMER, D. & HART, L.I. (1977). Reactivation of dihydrofolate reductase inhibited by methotrexate or aminopterin. *Arch. Biochem. Biophys.*, **182**, 646–656.

JACKSON, R.C., HART, L.I. & HARRAP, K.R. (1980). Intrinsic resistance to methotrexate of cultured mammalian cells in relation to the inhibition kinetics of their dihydrofolate reductase. *Cancer Res.*, **36**, 1991–1997.

JACKSON, R.C., FRY, D.W., BORITZKI, T.J., BESSERER, J.A., LEOPOLD, W.R., SLOAN, B.J. & ELSLAGER, R.F. (1984). Biochemical pharmacology of the lipophilic antifolate, trimetrexate. *Adv. Enz. Regul.*, **22**, 187–206.

KAMEN, B.A., EIBL, B., CASHMORE, A.R. & BERTINO, J.R. (1984). Uptake and efficacy of tremetrexate (TMQ, 2,4-diamino-5-methyl-6-[(3,4,5-tremethoxyanilino)methyl quinazoline], a non-classical antifolate in methotrexate-resistant leukemia cells in vitro. *Biochem. Pharmacol.*, **33**, 1697–1699.

KAUFMAN, R.J. & SCHIMKE, R.T. (1981). Amplification and loss of dihydrofolate reductase genes in a Chinese hamster ovary cell line. *Mol. Cell Biol.*, **1**, 1069–1076.

KLOHS, W.D., STEINKAMPF, R.W., BESSERER, J.A. & FRY, D.W. (1986). Cross resistance of pleiotropically drug resistant P388 leukemia cells to the lipophilic antifolates trimetrexate and BW301U. *Cancer Lett.*, **31**, 253–260.

KRISHAN, A. (1975). Rapid flow cytometric analysis of mammalian cell cycle by propidium iodide staining. *J. Cell Biol.*, **66**, 188–193.

LAEMMLI, U.K. (1970). Cleavage of structural protein during the assembly of the head of bacteriophage T4. *Nature*, **227**, 680–685.

LIN, J.T. & BERTINO, J.R. (1987). Trimetrexate, a second generation folate antagonist in clinical trial. *J. Clin. Oncol.*, **5**, 2032–2040.

MCMARTIN, K.E., NIRAYOTHA, V. & TEPHLY, T.R. (1981). High pressure liquid chromatography: separation and determination of rat liver folates. *Arch. Biochem. Biophys.*, **209**, 127–136.

MELERA, P.W., DAVIDE, J.P. & OEN, H. (1987). Antifolate-resistant Chinese hamster cells, molecular basis for the biochemical and structural heterogeneity among DHFRs produced by drug-sensitive and drug-resistant cell lines. *J. Biol. Chem.*, **262**, 1978–1990.

MINI, E., MOROSON, B.A., FRANCO, C.T. & BERTINO, J.R. (1985). Cytotoxic effects of folate antagonists against methotrexate-resistant human acute lymphoblast CCRF-CEM cell lines. *Cancer Res.*, **45**, 325–330.

MORAN, R.G., MULKINS, M. & HEIDELBERGER, C. (1979). Role of thymidylate synthetase activity in development of methotrexate cytotoxicity. *Proc. Natl Acad. Sci. USA*, **76**, 5924–5928.

MYERS, C.E., LIPPMAN, M.E., ELIOT, H.M. & CHABNER, B.A. (1975). Competitive protein binding assay for methotrexate. *Proc. Natl Acad. Sci. USA*, **72**, 3683.

PARK, J.G., OIE, H.K., SUGARBAKER, P.H., HENSLEE, J.G., CHEN, T.R., JOHNSON, B.E. & GAZDAR, A.F. (1987). Characteristics of cell lines established from human colorectal carcinoma. *Cancer Res.*, **47**, 6710–6718.

PIZZORNO, G., MINI, E., CORONNELLO, M., MCGUIRE, J.J., MOROSON, B.A., CASHMORE, A.R., DREYER, R.N., LIN, J.T., MAZZEI, T., PERITI, P. & BERTINO, J.R. (1988). Impaired polyglutamylation of methotrexate as a cause of resistance in CCRF-CEM cells after short-term, high-dose treatment with this drug. *Cancer Res.*, **48**, 2149–2155.

ROBERTS, D. (1966). An isotopic assay for thymidylate synthetase. *Biochemistry*, **5**, 3546–3548.

ROSS, E.D., CHEN, S.R.S. & CUDDY, D.P. (1990). Effects of 1-$\beta$-arabinofuranosylcytosine on DNA replication intermediates monitored by pH-step alkaline elution. *Cancer Res.*, **50**, 2658–2666.

SCHUETZ, J.D., MATHERLY, L.H., WESTIN, E.H. & GOLDMAN, I.D. (1988). Evidence for a functional defect in the translocation of the methotrexate transport carrier in a methotrexate-resistant L1210 leukemia cell line. *J. Biol. Chem.*, **263**, 9840–9847.

SHARMA, R.C., ASSARAF, Y.G. & SCHIMKE, R.T. (1991). A phenotype conferring selective resistance to lipophilic antifolates in Chinese hamster ovary cells. *Cancer Res.*, **51**, 2949–2959.

SWAIN, S.M., LIPPMAN, M.E., EGAN, E.F., DRAKE, J.C., STEINBERG, S.M. & ALLEGRA, C.J. (1989). Fluorouracil and high-dose leucovorin in previously treated patients with metastatic breast cancer. *J. Clin. Oncol.*, **7**, 890–899.

VAN DER VEER, L.J., WESTERHOF, G.R., RIJKSEN, G. & JANSEN, G. (1989). Cytotoxicity of methotrexate and trimetrexate and its reversal by folinic acid in human leukemic CCRF-CEM cells with carrier-mediated and receptor-mediated folate uptake. *Leukemia Res.*, **13**, 981–987.

VAN MOUWERIK, T.J., PANGALLO, C.A., WILLSON, J.K.V. & FISHER, P.H. (1987). Augmentation of methotrexate cytotoxicity in human colon cancer cells achieved through inhibition of thymidine salvage by dipyridamole. *Biochem. Pharmacol.*, **36**, 809–814.

WHITE, J.C. & GOLDMAN, I.D. (1981). Methotrexate resistance in an L1210 cell line resulting from increased dihydrofolate reductase, decreased thymidylate synthetase activity and normal membrane transport. *J. Biol. Chem.*, **256**, 5722–5727.

YALOWICH, J.C. & KALMAN, T.I. (1985). Rapid determination of thymidylate synthase activity and its inhibition in intact L1210 leukemia cells in vitro. *Biochem. Pharmacol.*, **34**, 2319–2324.